# Humic Polyelectrolytes Facilitate Rapid Microwave Synthesis of Silver Nanoparticles Suitable for Wound-Healing Applications

**DOI:** 10.3390/polym16050587

**Published:** 2024-02-21

**Authors:** Yu Zhang, Konstantin S. Larionov, Simeng Zhang, Nikita A. Sobolev, Andrey I. Konstantinov, Dmitry S. Volkov, Evgeniya V. Suslova, Vladimir E. Chernov, Anton I. Poloskov, Ruslan I. Glushakov, Irina V. Perminova

**Affiliations:** 1 Department of Chemistry, Lomonosov Moscow State University, Leninskie Gory 1-3, 119991 Moscow, Russia; zhangyu13051837552@gmail.com (Y.Z.); konstantin.larionov@chemistry.msu.ru (K.S.L.); zhangsimeng@mail.ru (S.Z.); n.a.sobolev@outlook.com (N.A.S.); konstant@org.chem.msu.ru (A.I.K.); dmsvolkov@gmail.com (D.S.V.); suslova@kge.msu.ru (E.V.S.); 2 Kirov Military Medical Academy, Academician Lebedev Street 6, 194044 Saint Petersburg, Russia; vechernov@mail.ru (V.E.C.); a.i.poloskov@gmail.com (A.I.P.); glushakoffruslan@yandex.ru (R.I.G.)

**Keywords:** microwave heating, natural polyelectrolytes, humic substances, silver nanoparticles, surface plasmon resonance, TEM

## Abstract

This article describes the one-pot microwave synthesis of silver nanoparticles (AgNPs) assisted with natural polyelectrolytes—humic substances (HS). The humic polyelectrolytes served both as chemical reductants for silver ions and as end-capping agents for AgNPs. Three commercially available sodium humates extracted from lignites and leonardite and one sodium fulvate isolated from natural brown water seeped through peat deposits were used in this study. The dynamics of the growth rate of AgNPs was characterised by UV–VIS spectroscopy by measuring the intensity of surface plasmon resonance at 420 nm. Transmission electron microscopy was used to characterise the size and morphology of AgNPs. Dynamic light scattering was used to determine size distributions of the synthesised AgNPs in the solutions. It was established that both conventional and microwave syntheses assisted with the coal humates produced small-size AgNPs in the range from 4 to 14 nm, with the maximum share of particles with sizes of (6 ± 2) nm by TEM estimates. The peat fulvate yielded much larger NPs with sizes from 10 to 50 nm by TEM estimates. DLS measurements revealed multimodal distributions of AgNPs stabilised with HS, which included both single NPs with the sizes from 5 to 15 nm, as well as their dominating aggregates with sizes from 20 to 200 nm and a smaller portion of extra-large aggregates up to 1000 nm. The given aggregates were loosely bound by humic polyelectrolyte, which prevented the coalescence of AgNPs into larger particles, as can be seen in the TEM images. The significant acceleration in the reaction time—a factor of 60 to 70—was achieved with the use of MW irradiation: from 240 min down to 210–240 s. The coal humate stabilised AgNPs showed antimicrobial properties in relation to *S. aureus.* A conclusion was made regarding the substantial advantages of microwave synthesis in the context of time and scaling up for the large-scale production of AgNP-HS preparations with antimicrobial properties suitable for external wound-healing applications.

## 1. Introduction

Humic substances (HSs) are natural polyelectrolytes with many unique physicochemical and biological properties [1,2,3]. They find applications in nanoscience as polyfunctional macroligands for the formation of nanoparticles and nanocomposites [4,5,6]. However, the reported applications, except for the systematically studied synthesis of iron oxohydroxides [7,8,9], are mostly sporadic and scarce. This is because of the extreme complexity of HS mixtures produced by oxidation from the plant biomacromolecules during stochastically driven humification processes. As a result, HSs are composed of both low- and high-molecular-weight components highly substituted with multiple carboxylic and phenolic groups. This causes their acidic character and polyelectrolytic properties. Due to the above-described complexity of HS compositions, they are operationally defined according to solubility in alkalis and acids [10]. HSs are alkali extracts of organic matter from fossil fuels (e.g., coal, peat), as well as from soils and sediments, which can make up to 90% of OM in the highly oxidised low-rank lignites (e.g., leonardite). HSs, in turn, could be segregated into two further classes: humic acids (HAs), which are not soluble at acidic conditions (pH < 2), and fulvic acids (FAs), which are soluble in the full pH range. In practice, they are isolated by the acidification of HS extracts until 2. The properties of HSs vary largely and are dependent both on the source of their origin and fractional composition. Coal HSs are the most hydrophobic among all other sources due to them having the highest content of aromatic fragments in the coal structure. They are mostly composed of aromatic acids and phenols. Coal HA fraction is enriched with phenols, whereas FA fraction is more acidic and dominated by carboxyl-substituted components. On contrary to the rather homogeneous aromatic structure of coal, the structure of peat is extremely heterogeneous due to the presence of both aromatic lignins and aliphatic polysaccharides. As a result, peat HSs are much more hydrophilic as compared to coal HSs, and peat FAs are the most hydrophilic among all other sources of HSs. Hence, if the application calls for material, which should be small, acidic, hydrophilic, highly charged, peat FAs should be selected; if the application calls for material with more complex, multidentate, phenolic, hydrophobic properties, then coal HAs should be considered.

Despite these substantial differences in the acid–base, redox, as well as the molecular weight properties of HS, which are completely defined by the source and fractionation procedure, they are often used interchangeably without a clear understanding of their specific properties. This undermines the quality of the obtained results and brings about the “bad flair” of HS applications: among the most frequent accusations are low transferability and reproducibility of the results, which kills all high-tech applications of HSs. We understand that reproducibility for technology is above benevolent properties and the lack of toxicity of HSs in the whole range of their concentrations in nature [3]. This is why the task of this research was to demonstrate the boundary conditions that make HS applications predictable and transferrable. For such a demonstration, we used the application of HSs for the synthesis of silver nanoparticles (AgNPs).

AgNPs are promising agents for suppressing resistant strains of pathogenic microorganisms, which makes them of particular importance for the healing of infected wounds [11,12,13]. Various studies have demonstrated that AgNPs can release Ag^+^ and generate reactive oxygen species for bacterial cell membrane destruction. They can also cause enzyme function disruption and DNA damage in bacteria, which ultimately leads to bacterial death [13,14,15,16,17]. There is a number of publications that show that HSs can be successfully used to reduce and stabilise silver NPs [18,19,20,21,22,23]. All reported studies used different sources and fractions of HSs. Alexandrova et al. [21,22] used coal HAs, mud Has, and shale HAs; Sal’nikov et al. [20] used peat FAs; and Litvin et al. [18,19] used artificial FAs. Our recent publication also presented screening results for a set of humic materials from different sources and fractional composition [23]. However, all reported studies so far used conventional heating technology [18,19,20,21,22,23]. This kind of synthesis is time- and labour-consuming. In addition, the shape and size of nanoparticles are difficult to control.

Microwave (MW)-assisted heating is of particular importance in this context. It has been widely used as an alternative to conventional heating for the synthesis of various polymers, inorganic compounds, nanoparticles, and biological nanomaterials [24,25,26]. Conventional heating tends to use a convective gradient to transfer heat from the surface to the reactor interior; moreover, the temperature is higher at the surface, which results in uneven heat distribution and reduces reaction yield [27]. High-energy MW irradiation can accelerate reactions, while modulation of its frequency may increase the selectivity of the formation of the desired reaction product. The use of MW heating offers quite a few advantages over conventional heating, such as instantaneous and rapid heating (deep inside heating), high temperature homogeneity, and selective heating [28]. MW-assisted heating has been also successfully used for the synthesis of AgNP [29,30,31,32]. However, there are only a few reports available on the synthesis of MW-assisted humics-based nanomaterials [33,34], but none of them are devoted to the synthesis of AgNPs.

Here, we present rapid a one-pot MW-assisted synthesis of AgNPs that was transferred from the conventional prototype. We used HSs from four different commercial sources to assess the reproducibility of the synthesis with regard to the quality of the final product—the size and antibacterial properties of AgNPs. Three similar humic products—sodium humates from coal extracted with alkali—were used for the purpose of this synthesis. We had to drop two more humates from the initial experimental set—they were manufactured with the use of sodium carbonate extraction instead of NaOH, which formed insoluble silver carbonate and prevented the formation of AgNPs. One more commercial product was used—sodium fulvate from peat—to add a structural gradient to the experimental sample set. The same conditions were applied with regard to the concentration of humic and silver precursors, as well as heating regime and isolation. A use of concentrated HS solutions (12 g/L) enabled the preparation of highly concentrated AgNPs of 40 mM. The sizes of AgNPs synthesised in the presence of all three sodium humates were smaller than 10 nm (6–7 nm), whereas much larger AgNPs were obtained in tne case of sodium fulvate at 20 nm. We provide data on the antimicrobial properties of the HS-stabilised AgNPs and a rationalisation of the prospects of their wound-healing applications.

## 2. Materials and Methods

### 2.1. Materials

Three sodium humates were used in this study: sodium humate extracted from lignite, which is marketed under the trade name “Relict” and produced by the Genesis LLC (Novosibirsk, Russia); sodium humate extracted from leonardite, which is marketed under the trademark Powhumus (Humintech GmbH, Grevenbroich, Germany); and sodium humate extracted from lignite, marketed under the trademark Life Force (Life Force Group LLC, Saratov, Russia). The humates were designated as CHG, CHP, and CHL, respectively. The sodium fulvate was extracted by ion exchange from water leachate of underground peat. It is marketed under the trademark Fulvagra (Humintech GmbH, Grevenbroich, Germany). Silver nitrate (Molychem (Mumbai, India), 98%) was used as a silver precursor, and sodium hydroxide (Kemphasol, Moscow, Russia, 95%) was purchased and used as it was.

The contents of elements (CHNS) in the humic materials used in this study were determined using a 2400 Series II CHNS/O elemental analyser (PerkinElmer, Waltham, MA, USA).

The structural group compositions were determined using ^13^C NMR spectroscopy. ^13^C NMR spectra were recorded in 0.3 M NaOD/D_2_O (99+% isotopic purity, Sigma Aldrich, St. Louis, MI, USA). The spectra were recorded on an Avance-400 NMR spectrometer (Bruker, Berlin, Germany) with a carrier frequency for ^13^C nuclei of 100 MHz. The INVGATE pulse sequence was used to exclude the nuclear Overhauser effect. A relaxation delay of 8 s was used for the complete relaxation of quaternary carbon atoms. To calculate the structure–group composition, the spectra were divided into nine intervals corresponding to the main structural components of HSs, and the intervals were integrated. The obtained integrals normalised to the whole spectrum represent the quantitative data of the structure–group composition of the studied HSs [35].

The content of silver in the final products was determined using an ICP-AES 720-ES spectrometer (Agilent Technologies, Santa Clara, CA, USA).

UV-visible absorption spectra were recorded using a UV/Vis Cary 50 Probe spectrometer (Varian, Palo Alto, CA, USA).

Transmission electron microscopy (TEM) images were obtained using a JEOL JEM-2100F microscope (JEOL, Akishima, Japan). For TEM investigation, a drop of the AgNPs-HS solution was placed onto a copper grid covered with a carbon film. The drop was brought to dryness (2 h under ambient air conditions), and the TEM image was detected at an accelerating voltage of 200 kV, a point resolution of 0.24 nm, and a line resolution of 0.18 nm. The processing of the image files was performed on more than 100 particles using the image analysis software “ImageJ 1.54d”.

Dynamic light scattering (DLS) experiments were carried out using Malvern Zetasizer Nano ZS (Worcestershire, UK). The measurements were performed on the AgNPs-HS samples obtained in this study after diluting them by a factor of 100 with a use of 0.2 M NaNO_3_ as a background electrolyte. The concentration of HSs was 120 mg/L, the concentration of Ag was 0.4 mM, and ionic strength was 0.2 M. The data treatment was performed using Malvern Software 8.01.

All reactions were carried out in a modified household microwave oven (Galanz MOG-2001M, Foshan, China).

### 2.2. Synthesis of Silver Nanoparticles

A weight of sodium humate (1 g) was dissolved in 35 mL of distilled water and centrifuged at 10,000 rpm for 10 min to remove any insoluble admixtures. The obtained supernatant was transferred into a 150 mL round-bottom flask, and 35 mL of deionised water was added. The pH of the obtained solution was adjusted to 11 using 1 M NaOH. Then, 15 mL of AgNO_3_ solution was added at a concentration of 38.7 g/L. The pH value of the obtained solution was adjusted to 10 using 1 M NaOH under constant stirring. The resulting reaction mixture had concentrations of AgNO_3_ and HSs of 6.8 and 11.7 g/L, respectively, which corresponded to 40 mM on the basis of metallic Ag in all the systems.

**Figure 1:**
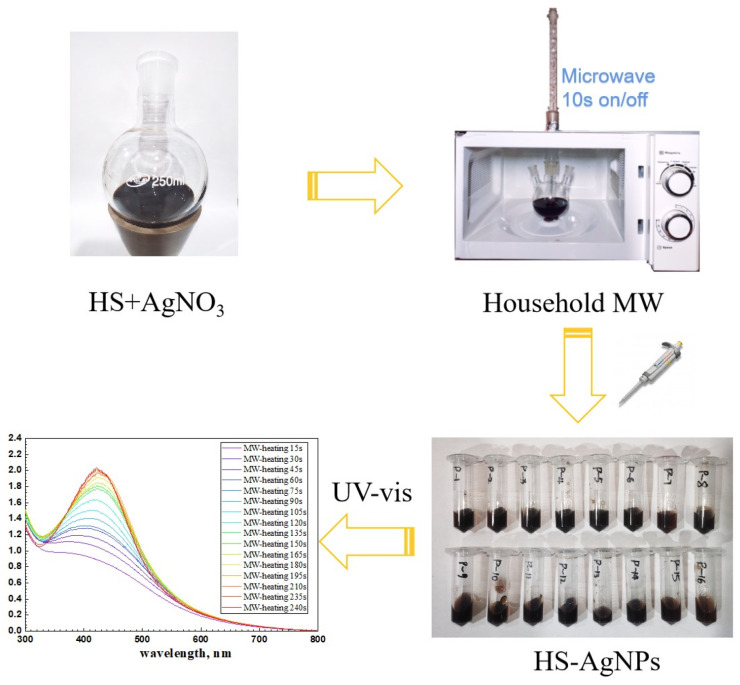
Scheme of microwave synthesis of HS-capped silver nanoparticles.

The microwave synthesis was conducted in a modified household microwave oven operating at 800 W using a sequence of pulses of 15 s and a pause of 10 s (10 s on/off) to prevent the solution from boiling and splashing due to overheating. The microwave exposure time was up to 240 s. After the completion of the synthesis, the samples were frozen and dried under vacuum. All syntheses were performed in the dark. The general scheme of microwave synthesis is shown in Figure 1.

For comparison, the synthesis of silver NPs by conventional heating was conducted with identical stoichiometries of the reagents in a foil-wrapped, three-necked, round-bottom glass bulb, which was placed into a water bath and heated under continuous stirring at 85 °C for 4 h.

### 2.3. Screening of Antimicrobial Activity of the Synthesised AgNP-HS Compositions

The antimicrobial tests of the clinical strain of Methicillin-resistant *Staphylococcus aureus* (MRSA) were carried out in 96-well plates using a non-agarised Lysogeny broth (LB) nutrient medium. For the tests, the required volume of 0.9% NaCl solution and 0.142 mL of LB medium were added to each well, and the corresponding amounts of the tested compositions of AgNP-HS with the initial concentrations of HSs of 11,700 μg/mL and 40 mM of AgNPs were added. The range of the resulting concentrations was from 0 to 1500 μg/mL for the concentration of HSs and from 0.7 to 5 mM (from 73 to 551 μg/mL) of AgNPs. MRSA colonies were pre-incubated at 35 °C in an unagarised Lysogeny broth (LB) for 12 h. A total of 0.025 mL of bacterial suspension was added to each well, which was brought to 2 IU according to McFarland and analysed on a Perkin Elmer Victor X5 multimode microplate reader (Perkin Elmer, Waltham, MA, USA) at a wavelength of 600 nm for 24 h.

## 3. Results and Discussion

### 3.1. Characteristics of Humic Materials Used in this Study

The humic materials used in this study were characterised with elemental analysis and ^13^C NMR spectroscopy. Elemental compositions are shown in Table 1, and structural group compositions are shown in Table 2.

The contents of elements were determined in the samples containing ash (about 20%). This is why the most informative data are the values of atomic ratios (H/C and C/N), which do not depend on the content of ash. The obtained values of the H/C ratio are indicative of much higher aromaticity of all three coal humates under study as compared to the fulvate from peat. This was expected, as peat contains polysaccharides and other aliphatic components.

**Figure 2:**
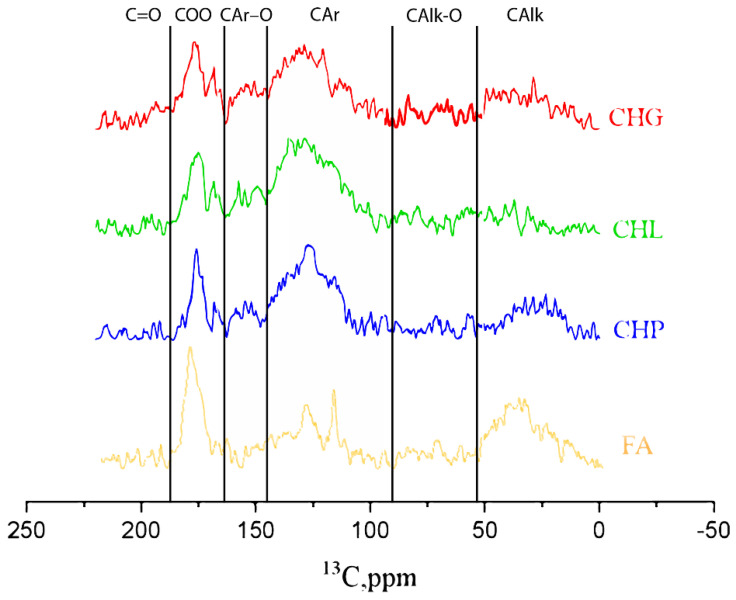
^13^C NMR spectra of the humic materials used in this study: sodium humates from leonaridte (CHP) and two lignites (CHG and CHL), and sodium fulvate isolated from peat DOM (FA).

The ^13^C NMR spectra are shown in Figure 2. The quantitative distribution of carbon among the main structural groups of the humic materials used in this study is provided in Table 1 in accordance with the carbon type assignments made in the obtained ^13^C NMR spectra.

The ^13^C NMR data are indicative of the dominating contribution of aromatic carbon in all three humates from coal, which is consistent with the highly aromatic character of their source material. The CHG sample was characterised with the highest contribution of aliphatic structures (36%) and the least content of aromatic carbon (47%), while the CHL sample demonstrated the highest content of aromatic carbon (60%) and the least content of aliphatics (20%). Both samples were extracted from lignite. The CHP sample had 55% and 30% of aromatic and aliphatic carbon, respectively. This sample was extracted from leonardite. The least content of aromatic carbon and the highest contents of “carbohydrate” (17%) and aliphatic (28%) carbons were observed for peat FA, which was consistent with the presence of polysaccharides in the source material of this HS. Given the found content of C in the humic materials used in this study (Table 1) and its distribution among the functional groups (Table 2), the concentrations of ArOH in the 11.8 g/L solutions of CHG, CHL, and CHP samples accounted for 34, 42, and 31 mM, respectively, and the FA solution contained 27 mM. The contents of the carboxylic groups in the same samples were 57, 65, 54, and 81 mM, respectively.

The data on elemental and structural-group composition of the humic materials used in this study demonstrated a drastic difference between the sodium fulvate from peat and three humates from coal. In addition, substantial variations in the content of aromatic carbon were observed among the three humates from coal.

### 3.2. Humic Polyanions—Assisted Synthesis of AgNPs under MW and Conventional Heating Conditions Followed by UV–VIS Spectroscopy

The reported values of the redox potential of HSs vary between 450 and 650 mV, which provides us its reducing properties with regard to Ag^+^ (E_0_ = 799 mV) [36]. The major reducing units are phenolic and hydroquinonic moieties. Given that humic polyanioins have both reducing (ArOH) and capping groups (COO-) in their structure, the used reagent amounts for all three Ag-HS systems yielded 1:1 to 1:2 ratios for Ag/ArOH stoichiometries depending on the use of one or two electron transitions by phenolic groups, as well as around 2:3 for Ag/COOH stoichiometries with regard to the amount of carboxylic groups. The excessive amounts of both reducing and complexing groups used in this study were to achieve a better conversion rate of ionic to metallic silver and the stabilisation of AgNPs. Formation of AgNPs in the HS solutions can be schematically described by the reaction (1):

AgNO_3_ + (ArOH)_HS_ + (COOH)_HS_ + 2OH^−^ = [Ag^0^(COO)_HS_] + ArO∙ + NO_3_^−^ + 2H_2_O (1)

Two-electron reduction of Ag^+^ ions by quinonoid-like moieties in humic polyanions can be accounted by the following reaction:(2)
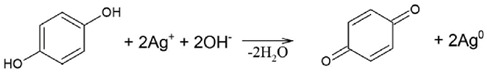


**Figure 3:**
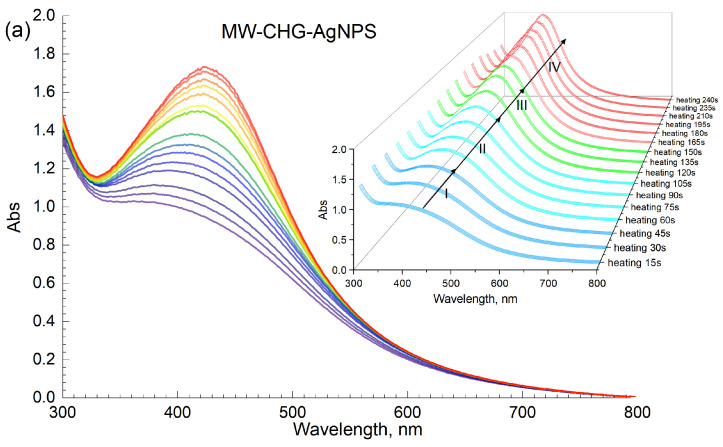

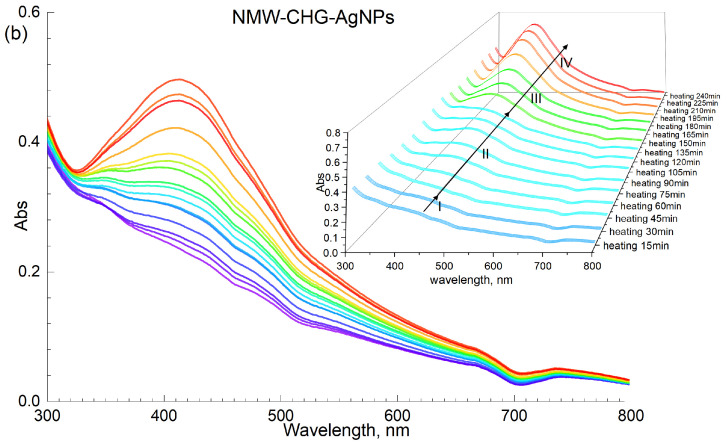
Synthesis of AgNPs mediated with HSs on the example of CHG sample: (**a**) microwave-assisted heating; (**b**) conventional water bath heating (85 °C); concentration of AgNPs at 40 mM, concentration of CHG at 11.8 g/L, pH 9. The Roman numbers from I to IV refer to the four stages of synthesis of AgNPs as described in the text.

The typical dynamics of UV–VIS spectra in the Ag-HS system under MW irradiation used in this study are shown in Figure 3a with the example of CHG-humate. Figure 3b shows the same system under conditions of conventional heating. Figure 3a shows a rapid growth in intensity of the surface plasmon resonance (SPR) peak of AgNPs located at 414 nm, along with an increase in the heating time. In the case of the MW-assisted heating, the SPR peak appeared already after the first 15 s of heating. The maximum value of the SPR intensity was reached after 4 min of MW irradiation: the longer treatment did not lead to the further increase in SPR intensity. This is indicative of the complete conversion of ionic silver species into metallic Ag^0^ over 4 min of MW heating.

**Figure 4:**
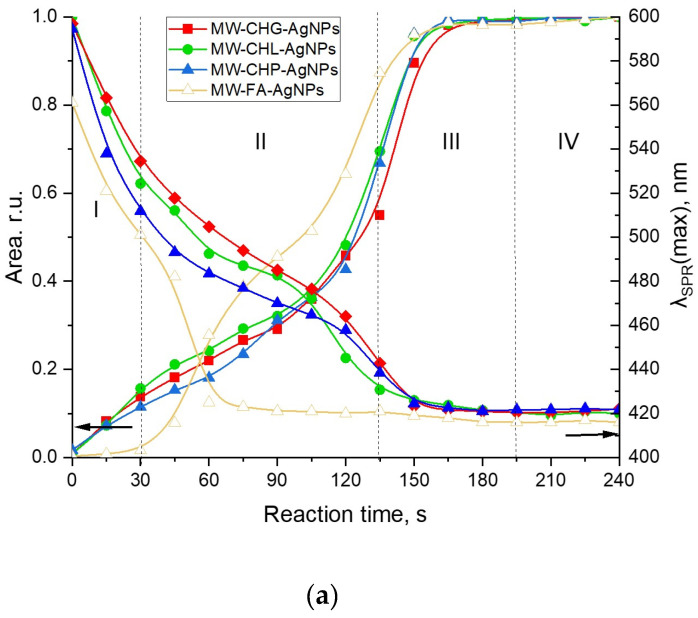

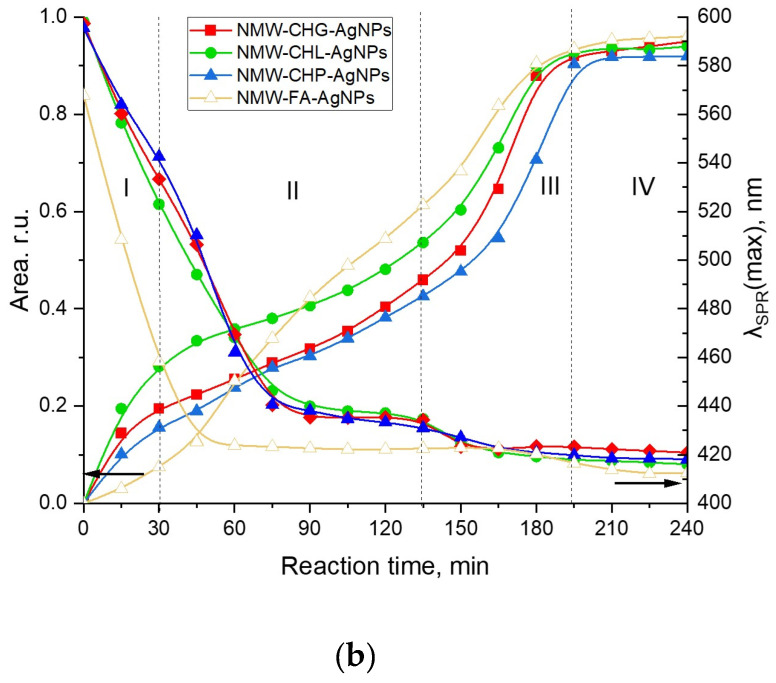
Evolution of the SPR peak of AgNPs during synthesis assisted with the four humic materials: CHG, CHL, CHP, and FAs under different heating conditions: (**a**) the MW-assisted heating; (**b**) the conventional heating at 85 °C. Concentrations: Ag—40 mM, sodium humate—11.8 g/L, pH 9; I (ionic strength) = 0.2 M. The Roman numbers from I to IV refer to the four stages of synthesis of AgNPs as described in the text. The left-headed black arrows point out to the dependences of Area of SPR on reaction time, the right-headed black arrows point out to the dependences of the SPR peak mosition (λ_SPR_(max)) on reaction time.

Figure 3b demonstrates the dynamics of UV–VIS spectra of the reaction mixture [AgNO_3_+HS] under conditions of conventional heating (85 °C). The formation of AgNPs occurred for several hours as compared to several minutes for the MW-assisted synthesis. The plots shown in Figure 3a, b demonstrate a much faster nucleation and growth of AgNP under microwave irradiation as compared to conventional heating. Evolution curves of the major SPR parameters, namely, the maximum wavelength and peak area for the four humic materials used in this study, are shown in Figure 4.

AgNP formation in the presence of concentrated solutions of different humic polyanions—humates and fulvates—differed substantially. At the same time, the SPR evolution curves for all three coal humates looked relatively similar. They were characterised by the steady initial growth of the AgNP population without any induction period, which was followed by a moderate slope and, finally, the steep slope of the curve. This type of growth kinetic curve was observed for both MW-assisted and conventional heating processes. At the same time, the evolution curve for the peat fulvate had an initial induction period, which was followed by a rapid growth of the SPR peak area, and then by a short intermediary stabilisation, followed by another very rapid growth period and a final stabilisation.

The initial stage of Ag^+^ interaction with the phenol-rich coal humates can be described as an ultrafast stage of reduction of Ag^+^ to Ag^0^ and nucleation into silver nanoclusters followed by a much slower process of AgNP aggregation (stage II) and, finally, by a fast process of coalescence to a final size of 5–7 nm (stage III) and the stable phase of constant AgNP population density in the solution (stage IV). The same stages for phenol-depleted but sugar-polyol-rich peat fulvate can be described as a slow initial process of reduction of Ag^+^ to Ag^0^ and nucleation into nanoclusters followed by very fast aggregation, much faster than in the case of the coal humates, with short stabilisation and a very fast coalescence up to the final particle size.

The character of the growth kinetic curves observed for the coal-humate-assisted synthesis of AgNPs was very similar to those reported by Polte et al. [37] and Van Hyning et al. [38]: they also lacked the induction stage. This type of growth curve does not obey the sigmoidal two-stage kinetic model for nucleation and the growth of noble metal nanoparticles, which was developed by the pioneering work of Watzky and Finke [39] that has been widely applied in the literature since [31,32,40,41]. At the same time, the latter model is more applicable to the peat FA.

### 3.3. AgNP Size Distribution Assessment Using TEM and DLS

**Figure 5:**
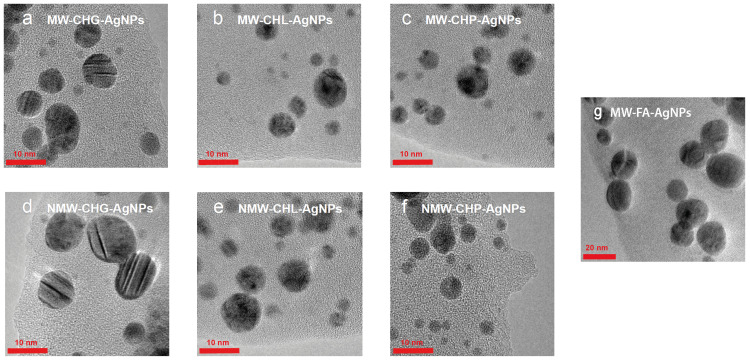
The particle size and distribution of AgNPs synthesised in the presence of three samples of coal humates used in this study under conditions of MW-assisted heating: (**a**) CHG, (**b**) CHL, (**c**) CHP, and under conditions of conventional heating: (**d**) CHG, (**e**) CHL, and (**f**) CHP. The AgNPs synthesised with MW irradiation in the presence of FAs are shown in (**g**).

For obtaining a better understanding of the sizes of the AgNPs formed and their distributions in the solutions containing high concentrations of HSs and salts, direct size measurements were undertaken using TEM, and the hydrodynamic radii of the particles in the solution were assessed using DLS. The TEM images of the AgNPs at the stable stage IV for all syntheses are shown in Figure 5.

**Figure 6:**
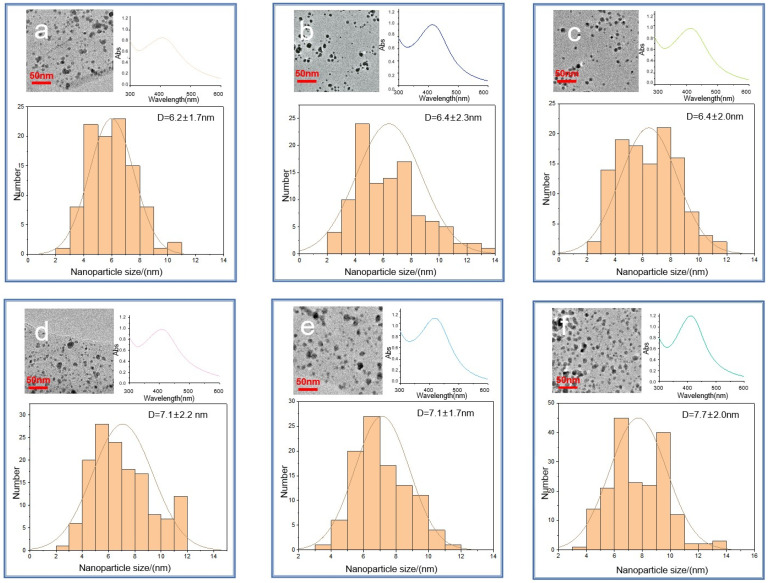
TEM images of the six Ag-HS systems used in this study obtained under MW irradiation (**a**–**c**) and conventional heating (**d**–**f**): (**a**) MW-CHG-AgNPs (6.2 ± 1.7) nm; (**b**) MW-CHL-AgNPs (6.4 ± 2.3) nm; (**c**) MW-CHP-AgNPs (6.4 ± 2.0) nm; (**d**) NMW-CHG-AgNPs (7.1 ± 2.2) nm; (**e**) NMW-CHL-AgNPs (7.1 ± 1.7) nm; (**f**) NMW-CHP-AgNPs (7.7 ± 2.0) nm. The histograms show the particle size distribution. The insets in the right upper corner show UV–VIS spectra at the final stage of synthesis.

The six Ag-HS systems prepared with the use of the higher-molecular-weight, phenolic-rich coal humates at the final synthesis stage yielded AgNPs under 10 nm with sizes ranging predominantly from 4 to 8 nm (Figure 5a–f), whereas AgNPs synthesised with a use of low-molecular-weight more aliphatic peat fulvate were much larger in size, ranging between 10 and 30 nm, with a predominant size of 20 nm (Figure 5g). The statistics of the obtained TEM data are shown in Figure 6.

The comparison of the size distributions of AgNPs obtained with a use of three coal humates under conditions of conventional heating and MW irradiation shows a lack of substantial differences among them: all coal humates showed high performance both in the reduction of ionic silver and in capping the formed AgNPs. Of particular importance for this study is the small size of the obtained Ag NPs, which varied from 2 to 12 nm, with distribution maxima located in the range from 4 to 8 nm. We did not observe a substantial reduction in size and polydispersity between AgNPs obtained with a use of conventional and MW heating, despite a much shorter synthesis time—4 min instead of 4 h.

**Figure 7:**
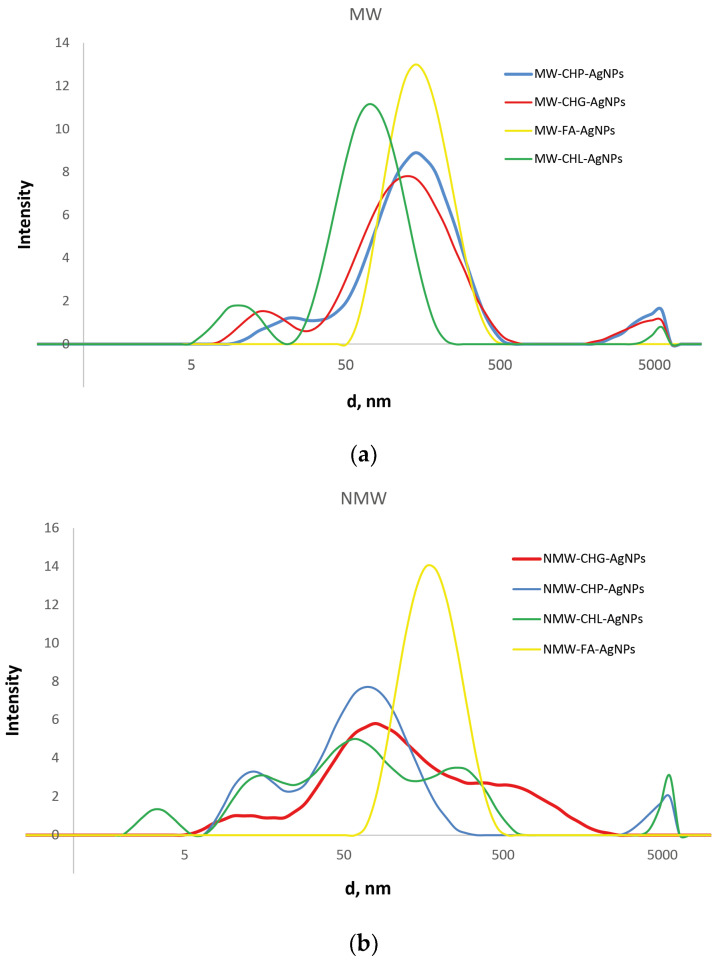
The distribution of hydrodynamic diameters of the particles present in the obtained AgNP-HS systems at the ionic strength of 0.2 M, as measured by DLS. The results are shown for the systems obtained with a use of MW irradiation (**a**) and conventional heating (**b**).

Given that TEM images do not provide realistic data on sizes of NPs in the solutions, we performed DLS measurements on all AgNP-HS systems used in this study. The systems were diluted down to a factor of 100 to account for the light absorption of HS. We kept the ionic strength of the solutions at the same value of 0.2 M by using sodium nitrate of this concentration as a dilutor. The obtained results are provided in Figure 7.

The data shown in Figure 7 reveal substantial differences in the size distribution of the three coal-humate-stabilised AgNPs from the peat-FA-stabilised AgNPs. While the former were characterised by multimodal broad distributions with sizes from 5 to 500–1000 nm, the latter had a much narrower monomodal distribution located between 50 and 500 nm with a maximum at around 200 nm. It should be noted that the both FA-stabilised AgNP systems had identical distribution, regardless of the type of heating—conventional or microwave. At the same time, for the coal humates, the modal distributions of the MW-assisted systems were much narrower as compared to the conventional heating. The three coal humates were characterised by the presence of narrow small-size components from 5 to 15 nm and broad medium-size components from 15 to 200 nm: the medium component had three modes for the conventional heating and monomodal structure for the MW heating. The CHG- and CHL-stabilised AgNPs had very similar broad-band distributions under conventional heating, whereas CHL-stabilised AgNPs in the case of MW synthesis had a single-band medium component with the smallest size located at around 50 nm. Quantitative characteristics of the obtained distributions (average sizes of the components) are summarised in Table 3.

The obtained DLS data are indicative of the presence in the solutions of AgNPs stabilised with the coal humates of a small portion of the single AgNPs with sizes between 5 and 15 nm, which corroborate well with the sizes seen on TEM images (Figure 5 and Figure 6). At the same time, a much larger portion of the AgNPs in solution was present in the form of loose aggregates bound together with humic macroligands. These aggregates had sizes from 20 to 200 nm, which were displayed as very intense broad peaks with shoulders reaching out up to 1000 nm in size. These shoulders were particularly distinct for CHG- and CHL-stabilised AgNPs. The values of zeta-potential measured for all systems showed good colloidal stability of all the systems under study (the values of potential were near or below 30 mV), with the highest values (below 40) found for CHG and FAs (Table 3).

The important issue is if the AgNPs stabilised with humic polyanions remain redispersible within aggregates observed in DLS curves. For answering this question, we conducted theoretical calculations of colloidal stability of the humics-stabilised AgNPs with a use of the model developed by our group for interactions of gold NPs stabilised with HSs [5]. As in the case of AuNPs, we considered the interaction of two identically charged spherical AgNPs with a radius *a*. We used the following expression for the total energy of interaction of colloidal NPs (*V_tot_*) expressed in kBT units (kB and T are the Boltzmann constant and absolute temperature, respectively):

(3) $$V_{tot}=V_{vdw}+V_{el-st}+V_{solv}$$

**Figure 8:**
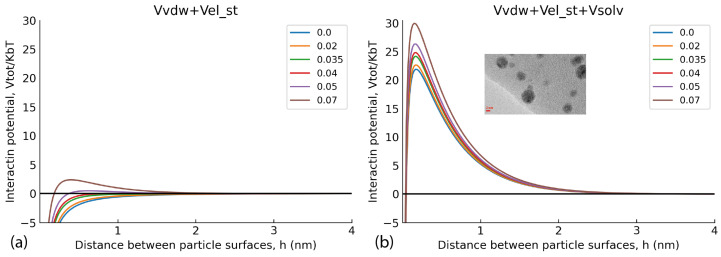
Pairwise AgNP interaction potentials at different values of surface potential ψ_0_. (**a**) the model calculations without consideration of repulsive solvation forces potential (V_solv_), (**b**) the interaction potentials calculated with the use of three terms including repulsive solvation forces.

The first term refers to attraction Van der Waals forces; the second term refers to electrostatic interactions—both terms are accounted for by the DLVO theory of colloidal stability of particles in the solution [42]. The third term refers to solvation forces, which are not accounted by the DLVO theory. The solvation forces account for the contribution of steric factors such as a mechanical barrier created by the sorbed polymer on the surface of a particle. For the sake of calculations, let *r* be the distance between centres of the interacting particles, and *h* = *r* − 2*a* is the shortest distance between their surfaces. The attraction potential (*V_vdW_*) is given by the Hamaker expression for spherical particles [43]—we used the reported value of the silver/water/silver Hamaker constant *A* = 4 × 10^−20^ J [38]. The Ohshima expression [44,45,46] was used for the calculation of *V_el−st_*, since the condition *a*/*r_D_* < 5 was fulfilled. In this study the ratio *a/r_D_* was 4.8, while the particle radius was 3 nm (estimated from the TEM results in Figure 5), and *r_D_* (the Debye radius) was equal to 0.63 nm for the ionic strength of 0.2 M used in our studies. The Bjerrum length *l_b_* was set to 0.6 nm because the syntheses were performed at *T* = 350 K. Parameters for the calculation of solvation force potential were: *l* = 0.5 nm and *B*_s_ = 2.2 × 10^−2^ J∙m^−2^. We paid particular attention to solvation forces while at the distances under few nanometres they may substantially contribute to interparticle interaction and provide stability of the dispersion. In the published model [5], the steric repulsion of soft adsorbed layers was approximated by a smooth repulsive potential *V_solv_* instead of the infinite vertical potential wall, which was used by Chou and Zukoski [47]. Thus, the solvation repulsive forces potential can be written in the framework of Derjaguin approximation (h ≪ a) [42] as follows:

(4) $$V_{solv}=2\pi \frac{B_{s}al}{k_{B}T}exp\left(-\frac{h}{l}\right)$$

with the decay length *l* = 0.5 nm and parameter *B_s_* = 22 × 10^−3^ J m^−2^. Full mathematical expressions are given in the source publication by Polyakov et al. (2017) [5] in the ESI. The model was applied for the qualitative explanation of how particle surface potential, which increases along with a sorption of humic polyanions at the surface of NP, affects the dispersion stability. Figure 8a shows a set of pairwise interaction potentials that corresponded to different ψ_0_ values (in *V*) for the case when only Van der Waals attractive forces and electrostatic repulsion was accounted for (two terms of Equation (3)), and Figure 8b shows the same set of interaction potentials calculated with the use of three terms, which took care of repulsive solvation forces.

Figure 8a shows that under the used experimental conditions (high ionic strength 0.2 M and small Debye radius), a use of the two-term interaction model prognosticated irreversible flocculation of nanoparticles—all curves were below 0, which contradicted experimental data. At the same time, incorporation of the third term responsible for steric repulsion yielded very different results. The curves of interaction potential in Figure 8b show that at all values of interaction potential, AgNPs would coalesce only when approaching each other by less than 0.4 nm. Otherwise, the potential barrier was too large to allow the AgNPs to irreversibly aggregate. The sorbed layer of humic polyanions prevents such a close proximity of the particles. This corroborates the TEM data (Figure 6)—the particles existed in the dispersed state due to thermal motion of particles. So, the conceptual results of the modelling indicate the reversible character of the aggregates of AgNPs stabilised with HS, which was able to be observed in the DLS curves. They can redisperse under conditions of smaller ionic strength.

In total, the obtained results demonstrate that the use of MW irradiation allowed us to reduce the synthesis time from 240 min down to 240 s, or more precisely, to 210 s; there was no change observed in the system during the last 30 s of heating. So, the MW-assisted synthesis proceeded by a factor of 60 to 70 faster as compared to the conventional heating one. It should also be noted that the use of coal humates turned out to be much more preferential for achieving smaller sizes of AgNPs as compared to peat fulvate. This could be connected both to different reducing groups present in the structure of fulvates and humates, as well as to the higher molecular weights of humates versus fulvates. Given both the smaller sizes of AgNPs, stabilised with the coal humates, and the much higher availability of coal humates on the market, antibacterial activity was tested only for six coal-humate-stabilised AgNPs.

### 3.4. Assessment of the Antibacterial Activity of the AgNPs Stabilised with Coal Humates Synthesised in This Study

**Figure 9:**
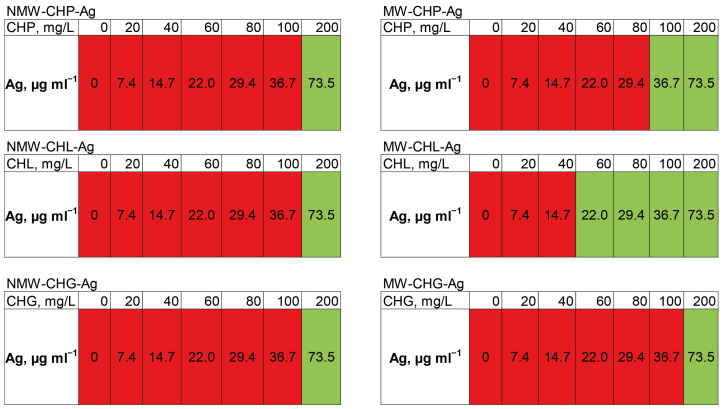
The change in optical density in 24 h when measuring the minimum inhibitory concentration of the synthesised compositions of AgNP-HS in the LB medium with the concentrations of HSs from 0 to 200 mg/L shown in the upper raw of the tables and concentration of AgNPs from 0 to 73.5 mg/L in relation to the clinical strain of MRSA. Green indicates complete suppression of bacterial growth, and red indicates the absence of inhibition.

The antibacterial effects of the compositions of AgNPs synthesised in the presence of coal humates were studied in relation to the methicillin-resistant strain of *Staphylococcus aureus* (MRSA). The suppression of the growth of this clinical strain was tested with the six AgNP-HS compositions in the concentration range of HSs from 0 to 200 mg/L and of AgNPs from 0 to 73.5 mg/L. The results on minimum inhibitory concentration (MIC) determination for the AgNPs under study are shown in Figure 9.

It can be seen that the silver concentration which suppressed bacterial growth (shown in green in Figure 9) upon the addition of all six compositions accounted for 73.5 mg/L. The highest antibacterial activity—the lowest MIC—was observed for MW-CHL-Ag composition: it accounted for 22 mg/L of Ag. It should be noted that this composition was characterised with the smaller size AgNP aggregates as compared to CHG and CHP (Figure 7b). The obtained MIC values lay within those reported for other AgNP-based compositions against MRSA strains [14]. In total, the obtained data provide a good promise for wound applications of AgNP-HS compositions. Further studies are planned to reduce the size of AgNPs stabilised with HSs and to optimise MICs of the AgNP-HS compositions.

## 4. Conclusions

This contribution reports for the first time the results of feasibility studies on the MW-assisted synthesis of concentrated solutions (40 mM) of small-size AgNPs in the presence of natural humic polyelectrolytes. The specific goal of this study was to develop rapid, large-scale, high-yield synthesis of AgNPs of high antibacterial activity suitable for wound-healing applications. For achieving this goal, we used MW irradiation as a source of energy to accelerate both the reduction rate of ionic silver to metallic particles and its growth rate until a final size of the particles in the range from 4 to 10 nm. This brought about a factor of 60 to 70 in in synthesis time: we were able to shorten it down to 3–4 min instead of 240 min under conditions of conventional heating with a use of a water bath. We used three commercially available sodium humates extracted from coal and one peat fulvate as the solo reducing and capping agents for producing AgNPs. The use of humic materials has several advantages: First of all, by their beneficial biological profile: they are natural polyfunctional polyelectrolytes which do not pose toxic effects to living organisms in the whole range of their natural concentrations. In addition, they possess pronounced antioxidant activity due to enrichment with phenolic groups. We have recently reported that combinations of HSs with AgNPs [23] bring about enhanced antioxidant effects and reduced cytotoxicity. The commercial humates are reasonably priced and provide a rich source of widely available natural polyelectrolytes suitable for biomedical applications. A direct comparison of the coal humates with peat fulvates has demonstrated an influence of the source of humic material and a technique of its isolation on the molecular weight and structural group composition of the isolated humic materials. The obtained results are very promising for the use of coal-humate-based AgNP compositions in wound-healing applications, which are very demanding both with regard to the concentration of the antibacterial agent—metallic Ag—in the medication and regarding the amount of medication needed for the efficient clinical treatment of infected wounds because of the large size and slow healing process of infected wounds.

## 5. Patents

The patent application “Preparation of antibacterial compositions of AgNPs capped with HS” is underway in its submission to the Russian Patent Office. It results from the work reported in this manuscript.

## Figures and Tables

**Table 1:** Elemental compositions of the humic materials used in this study.

	Content of Elements, % (Mass Including Ash)				Atomic Ratios	
Sample	C	H	N	S	H/C	C/N
CHL	39.2 ± 0.2	3.35 ±0.05	0.97 ±0.03	0.48 ± 0.04	1.0	47
CHP	39.6 ±0.5	3.62 ±0.08	0.91 ± 0.03	0.51±0.01	1.1	51
CHG	38.5 ± 0.3	3.78 ± 0.04	0.76 ± 0.01	0.47 ± 0.03	1.2	59
FA	40.2 ± 0.1	4.29 ± 0.03	0.70 ± 0.01	0.67 ± 0.01	1.3	67

**Table 2:** Carbon distribution in the humic materials used in this study (%) (^13^C NMR data).

Samples				Chemical Shift, ppm			
	0–56	56–89	89–145	145–165	165–185	185–220	
	C_Alk_	C_AlkO_	C_Ar_	C_ArO_	COO	C=O	ΣC_Ar_/ΣC_Alk_
CHL	26	10	38	9	15	2	1.3
CHP	9	11	49	11	17	3	3.0
CHG	21	8	47	8	14	3	1.9
FA	28	17	22	7	21	5	0.63

**Table 3:** The average values of sizes (d) and zeta-potential (Z) for eight AgNP-HS systems used in this study (ionic strength—0.2 M; concentration of HS—118 mg/L; concentration of Ag—0.4 mM).

Sample	d-Avg *, nm	d-Pk1-Avg *, nm	d-Pk2-Avg *, nm	d-Pk3-Avg *, nm	Z-Potential, mV
NMW-CHG-AgNPs	53	141	3	2918	−(44 ± 12)
NMW-CHP-AgNPs	87	177	2861	1	−(27 ± 19)
NMW-CHL-AgNPs	20	34	1912	1	−(30 ± 14)
NMW-FA-AgNPs	128	263	17	4612	−(43 ± 17)
MW-CHG-AgNPs	80	146	3352	6	−(35 ± 19)
MW-CHP-AgNPs	27	59	3	3500	−(28 ± 24)
MW-CHL-AgNPs	58	124	12	1	−(34 ± 29)
MW-FA-AgNPs	137	233	29	3645	−(36 ± 18)

* d-Avg refers to the average particle size of the whole distribution; d-Pk1, d-Pk2, and d-Pk3 refer to the average particle size of the main, second, and third peaks of the distributions shown in Figure 7, respectively.

## Data Availability

All data resulting from this study are provided in this manuscript.

## References

[1] Olk D.C., Bloom P.R., Perdue E.M., McKnight D.M., Chen Y., Farenhorst A., Senesi N., Chin Y.-P., Schmitt-Kopplin P., Hertkorn N. (2019). Environmental and Agricultural Relevance of Humic Fractions Extracted by Alkali from Soils and Natural Waters. J. Environ. Qual..

[2] Havelcová M., Mizera J., Sýkorová I., Pekar M. (2009). Sorption of metal ions on lignite and the derived humic substances. J. Hazard. Mater..

[3] Perminova I.V. (2019). From green chemistry and nature-like technologies towards ecoadaptive chemistry and technology. Pure Appl. Chem. PAC.

[4] Baigorri R., García-Mina J.M., Aroca R.F., Alvarez-Puebla A.A. (2008). Optical Enhancing Properties of Anisotropic Gold Nano-plates Prepared with Different Fractions of a Natural Humic Substance. Chem. Mater..

[5] Polyakov A.Y., Lebedev V.A., Shirshin E.A., Rumyantsev A.M., Volikov A.B., Garshev A.V., Goodilin E.A., Perminova I.V. (2017). Non-classical growth of water-redispersible spheroidal gold nanoparticles assisted by leonardite humate. CrystEngComm.

[6] Venezia V., Verrillo M., Gallucci N., Di Girolamo R., Luciani G., D’Errico G., Paduano L., Piccolo A., Vitiello G. (2023). Ex-ploiting bioderived humic acids: A molecular combination with ZnO nanoparticles leads to nanostructured hybrid interfaces with enhanced pro-oxidant and antibacterial activity. J. Environ. Chem. Eng..

[7] Pomogailo A.D., Kydralieva K.A., Zaripova A.A., Muratov V.S., Dzhardimalieva G.I., Pomogailo S.I., Golubeva N.D., Jorobekova S.J. (2011). Magnetoactive Humic-Based Nanocomposites. Macromolecular Symposia.

[8] Illés E., Tombácz E. (2006). The effect of humic acid adsorption on pH-dependent surface charging and aggregation of magnetite nanoparticles. J. Colloid Interface Sci..

[9] Kulikova N.A., Polyakov A.Y., Lebedev V.A., Abroskin D.P., Volkov D.S., Pankratov D.A., Klein O.I., Senik S.V., Sorkina T.A., Garshev A.V. (2017). Key roles of size and crystallinity of nanosized iron hydr(oxides) stabilized by humic substances in iron bioavailability to plants. J. Agric. Food Chem..

[10] Stevenson F.J. (1994). Humus Chemistry.

[11] Anees A., Sachi Das S., Khatoon A., Tahir Ansari M. (2020). Bactericidal Activity of Silver Nanoparticles: A Mechanistic Review. Mater. Sci. Energy Technol..

[12] El-Aassar M.R., Ibrahim O.M., Fouda M.M.G., El-Beheri N.G., Agwa M.M. (2020). Wound Healing of Nanofiber Comprising Polygalacturonic/Hyaluronic Acid Embedded Silver Nanoparticles: In-Vitro and In-Vivo Studies. Carbohydr. Polym..

[13] Paladini F., Pollini M. (2019). Antimicrobial Silver Nanoparticles for Wound Healing Application: Progress and Future Trends. Materials.

[14] Swolana D., Wojtyczka R.D. (2022). Activity of Silver Nanoparticles against *Staphylococcus* spp.. Int. J. Mol. Sci..

[15] Slavin Y.N., Asnis J., Hńfeli U.O., Bach H. (2017). Metal nanoparticles: Understanding the mechanisms behind antibacterial activity. J. Nanobiotechnol..

[16] Deng H., McShan D., Zhang Y., Sinha S.S., Arslan Z., Ray P.C., Yu H. (2016). Mechanistic study of the synergistic antibacterial activ-ity of combined silver nanoparticles and common antibiotics. Environ. Sci. Technol..

[17] Wang E., Huang Y., Du Q., Sun Y. (2017). Silver Nanoparticle Induced Toxicity to Human Sperm by Increasing ROS (Reactive Oxygen Species) Production and DNA Damage. Environ. Toxicol. Pharmacol..

[18] Litvin V.A., Galagan R.L., Minaev B.F. (2012). Kinetic and Mechanism Formation of Silver Nanoparticles Coated by Synthetic Humic Substances. Colloids Surf. A Physicochem. Eng. Asp..

[19] Litvin V.A., Minaev B.F. (2013). Spectroscopy study of silver nanoparticles fabrication using synthetic humic substances and their antimicrobial activity. Spectrochim. Acta Part A Mol. Biomol. Spectrosc..

[20] Sal’nikov D.S., Pogorelova A.S., Makarov S.V., Vashurina I.Y. (2009). Silver ion reduction with peat fulvic acids. Russ. J. Appl. Chem..

[21] Aleksandrova G.P., Lesnichaya M.V., Dolmaa G., Klimenkov I.V., Sukhov BGRegdel D., Trofimov B.A. (2017). Silver-containing nanocomposites with antioxidant activity based on humic substances of different origin. Russ. Chem. Bull. Int. Ed..

[22] Alexandrova G.P., Dolmaa G., Enkhbadral U., Grishenko G.L., Tserenpi S., Sukhov B.G., Regdel D., Trofimov B.A. (2012). A new humic acid remedy with addition of silver nanoparticles. Mong. J. Chem..

[23] Zykova M.V., Volikov A.B., Buyko E.E., Bratishko K.A., Ivanov V.V., Konstantinov A.I., Logvinova L.A., Mihalyov D.A., Sobolev N.A., Zhirkova A.M. (2023). Enhanced Antioxidant Activity and Reduced Cytotoxicity of Silver Nanoparticles Stabilized by Different Humic Materials. Polymers.

[24] Bartoli M., Frediani M., Briens C., Berruti F., Rosi L. (2019). An Overview of Temperature Issues in Microwave-Assisted Pyrolysis. Processes.

[25] Gawande M.B., Shelke S.N., Zboril R., Varma R.S. (2014). Microwave-Assisted Chemistry: Synthetic Applications for Rapid Assembly of Nanomaterials and Organics. Acc. Chem. Res..

[26] Sun J., Wang W., Yue Q. (2016). Review on Microwave-Matter Interaction: Fundamentals and Efficient Microwave-Associated Heat-ing Strategies. Materials.

[27] Liu Q., Gao M.R., Liu Y., Okasinski J.S., Ren Y., Sun Y. (2016). Quantifying the Nucleation and Growth Kinetics of Microwave Nanochemistry Enabled by in Situ High-Energy X-Ray Scattering. Nano Lett..

[28] Praneeth N.V.S., Paria S. (2018). Microwave-Assisted One-Pot Synthesis of Anisotropic Gold Nanoparticles with Active High-Energy Facets for Enhanced Catalytic and Metal Enhanced Fluorescence Activities. CrystEngComm.

[29] Nishioka M., Miyakawa M., Kataoka H., Koda H., Sato K., Suzuki T.M. (2011). Continuous Synthesis of Monodispersed Silver Nanoparticles Using a Homogeneous Heating Microwave Reactor System. Nanoscale.

[30] Helmlinger J., Heise M., Heggen M., Ruck M., Epple M. (2015). A Rapid, High-Yield and Large-Scale Synthesis of Uniform Spherical Silver Nanoparticles by a Microwave-Assisted Polyol Process. RSC Adv..

[31] Thanh N.T.K., Maclean N., Mahiddine S. (2014). Mechanisms of Nucleation and Growth of Nanoparticles in Solution. Chem. Rev..

[32] Özkar S., Finke R.G. (2017). Silver Nanoparticles Synthesized by Microwave Heating: A Kinetic and Mechanistic Re-Analysis and Re-Interpretation. J. Phys. Chem. C.

[33] Kostyukhin E.M., Nissenbaum V.D., Abkhalimov E.V., Kustov A.L., Ershov B.G., Kustov L.M. (2020). Microwave-Assisted Synthesis of Water-Dispersible Humate-Coated Magnetite Nanoparticles: Relation of Coating Process Parameters to the Properties of Nanoparticles. Nanomaterials.

[34] da Silva L.C., Abate G., Oliveira N.A., Fantini M.C.D.A., Masini J.C., Mercuri L.P., Olkhovyk O., Jaroniec M., Matos J.R. (2005). Microwave synthesis of FDU-1 silica with incorporated humic acid and its application for adsorption of Cd^2+^ from aqueous solutions. Stud. Surf. Sci. Catal..

[35] Hertkorn N., Permin A., Perminova I., Kovalevskii D., Yudov M., Petrosyan V., Kettrup A. (2002). Comparative Analysis of Partial Structures of a Peat Humic and Fulvic Acid Using One- and Two-Dimensional Nuclear Magnetic Resonance Spectroscopy. J. Environ. Qual..

[36] Cao X., Ma C., Zhao J., Guo H., Dai Y., Wang Z., Xing B. (2019). Graphene oxide mediated reduction of silver ions to silver nanoparticles under environmentally relevant conditions: Kinetics and mechanisms. Sci. Total Environ..

[37] Polte J., Xenia Tuaev X., Wuithschick M., Fischer A., Thuenemann A.F., Rademann K., Kraehnert R., Emmerling F. (2012). For-mation Mechanism of Colloidal Silver Nanoparticles: Analogies and Differences to the Growth of Gold Nanoparticles. ACS Nano.

[38] Van Hyning D.L., Klemperer W.G., Zukoski C.F. (2001). Silver Nanoparticle Formation: Predictions and Verification of the Ag-gregative Growth Model. Langmuir.

[39] Watzky M.A., Finke R.G. (1997). Transition Metal Nanocluster Formation Kinetic and Mechanistic Studies. A New Mechanism When Hydrogen Is the Reductant: Slow, Continuous Nucleation and Fast Autocatalytic Surface Growth. J. Am. Chem. Soc..

[40] Finney E.E., Finke R.G. (2008). The Four-Step, Double-Autocatalytic Mechanism for Transition-Metal Nanocluster Nucleation, Growth, and Then Agglomeration: Metal, Ligand, Concentration, Temperature, and Solvent Dependency Studies. Chem. Mater..

[41] Xiong Y., Siekkinen A.R., Wang J., Yin Y., Kim M.J., Xia Y. (2007). Synthesis of Silver Nanoplates at High Yields by Slowing Down the Polyol Reduction of Silver Nitrate with Polyacrylamide. J. Mater. Chem..

[42] Israelachvili J.N. (1992). Intermolecular and Surface Forces.

[43] Hamaker H.C. (1937). The London—Van der Waals attraction between spherical particles. Physica.

[44] Ohshima H. (1995). Effective surface potential and double-layer interaction of colloidal particles. J. Colloid Interface Sci..

[45] Lee K., Sathyagal A.N., McCormickLee A.V. (1998). A closer look at an aggregation model of the Stöber process. Colloids Surf. A.

[46] Kim T., Lee K., Gong M.-S., Joo S.-W. (2005). Control of gold nanoparticle aggregates by manipulation of interparticle interaction. Langmuir.

[47] Chow M.K., Zukoski C.F. (1994). Gold sol formation mechanisms: Role of colloidal stability. J. Colloid Interface Sci..

